# Designing Exhibits to Support Relational Learning in a Science Museum

**DOI:** 10.3389/fpsyg.2021.636030

**Published:** 2021-03-26

**Authors:** Benjamin D. Jee, Florencia K. Anggoro

**Affiliations:** ^1^Department of Psychology, Worcester State University, Worcester, MA, United States; ^2^Department of Psychology, College of the Holy Cross, Worcester, MA, United States

**Keywords:** relational learning, science museum, comparison, informal learning, cognitive support

## Abstract

Science museums aim to provide educational experiences for both children and adults. To achieve this goal, museum displays must convey scientifically-relevant relationships, such as the similarities that unite members of a natural category, and the connections between scientific models and observable objects and events. In this paper, we explore how research on comparison could be leveraged to support learning about such relationships. We describe how museum displays could promote educationally-relevant comparisons involving natural specimens and scientific models. We also discuss how these comparisons could be supported through the design of a display—in particular, by using similarity, space, and language to facilitate relational thinking for children and their adult companions. Such supports may be pivotal given the informal nature of learning in museums.

## Introduction

Science museums aim to provide visitors with education as well as entertainment. In this paper, we consider how museum exhibits could be designed to promote cognitive processes that are instrumental to science learning. We focus on the process of *comparison*—a powerful mechanism that applies to a wide range of topics. We discuss how museum exhibits can promote comparisons to educate and engage visitors, and describe cognitive supports for comparison that are applicable to museums and other informal learning contexts.

## Relational Thinking in a Science Museum

Science learning involves *relational* thinking. For example, understanding the scope and boundaries of natural categories involves recognizing how members of a category are similar to one another and distinct from members of other groups. There are also the deeper evolutionary relationships between organisms that shed light on the process of natural selection. Many other scientific categories are defined by abstract relations, having few (if any) overt features in common (Richland and Simms, 2015; Goldwater and Schalk, 2016). For example, the ripening of a banana bears little resemblance to the melting of arctic permafrost, yet both share the abstract structure of a positive feedback system, a process perpetuated by its own effects. Relational thinking is also involved in learning about scientific models, which represent key properties and relationships inherent to an object or system (Clement, 2008; Sibley, 2009; Kastens and Rivet, 2010; Stull and Hegarty, 2016). Comprehending these models involves understanding the spatial, temporal, and causal structure that is represented, as well as the relationship between the model and the real world (Jee and Anggoro, 2019).

A key question is how to promote scientifically-relevant relational thinking in the context of the museum. We propose a general approach based on cognitive and educational research on relational thinking—namely, the use of *comparison*.

## Comparison Promotes Relational Thinking

Comparison involves aligning the elements of two representations according to the role they play in a common system of relations—a process of *structural alignment* (Gentner, 1983). For example, in the comparison between the atom (the less-familiar “target” case) and the solar system (the more-familiar “base”), structural relations, like the orbiting of smaller objects around a larger central object, are brought into focus. Superficial features, like the absolute size of the objects involved, fade into the background. Thus, the nucleus of the atom is placed in correspondence with the Sun—not because they look alike, but because these objects occupy a central position and attract surrounding objects in their respective systems. Through comparison, the broader relational structure that unites the two cases—that of a central force system—can be abstracted, forming a new relational concept (Gentner and Smith, 2012; Goldwater and Schalk, 2016). However, if the two cases are not explicitly compared, such abstractions may go unrealized (Gick and Holyoak, 1983; Kurtz et al., 2001; Richland et al., 2007; Star and Rittle-Johnson, 2009; Goldwater and Gentner, 2015).

Comparison is a powerful mechanism that has been used to meet a broad range of educational goals (Richland et al., 2007; Rittle-Johnson and Star, 2007; Alfieri et al., 2013; Jee et al., 2013). Comparison has also proven effective across a range of age levels, enhancing young children's and infants' relational thinking in a variety of domains (Gentner, 2010; Hespos et al., 2020). In the present paper, we explore how museum displays could be designed to promote scientifically-informative comparisons involving widely-used materials: natural specimens and scientific models.

We first consider how pairs of specimens could be used to promote learning of critical category information, including within-category variability, category distinctions, and shared structure that points to deep evolutionary relations. We then turn to learning about real-world causal systems through scientific models, and consider how pairing a model with a second, related representation could clarify the relationship between the model and the real world, and facilitate analogical reasoning about unfamiliar causal systems.

When it comes to museum-based learning—which is more self-directed and less structured than formal instruction (Hurst et al., 2019)—it cannot be taken for granted that visitors will engage in relevant comparisons, even when an informative pair of items is presented in a display. Nor can it be assumed that children's accompanying caregivers will provide appropriate assistance. In fact, parents can be unmotivated to provide an “educational experience” for their children (Collaboration for Ongoing Visitor Experience Studies (COVES), 2018), and may underestimate the educational value of museum exhibits (Song et al., 2017). Methods that promote children's learning, and encourage adults' involvement in this learning, are crucial in this setting (Pattison and Bailey, 2016). Thus, we also consider how aspects of an exhibit display—including the visual appearance of the specimens or models in the display, how the display is structured, and how the display is described in surrounding signs, labels, and captions—could be designed to facilitate the structural alignment process for visitors.

## Exhibits that Promote Comparisons Involving Natural Specimens

Natural specimens—skeletons, fossils, rocks, shells, etc.—are a hallmark of science museums. These objects provide visitors with the opportunity to observe the diversity of life on Earth, a central aim of current science education frameworks, such as the Next Generation Science Standards (National Research Council, 2012, 2013). Exposure to natural specimens could also help to offset the “taxonomic impediment” identified by the Convention on Biological Diversity—i.e., the decline in taxonomic expertise, resources, and public and policy-maker awareness (e.g., Klopper et al., 2002). Hence, the effective display of natural objects is of central importance to a museum's educational goals.

Natural specimens are displayed in a variety of ways, from crowded display cases to large-scale dioramas that reconstruct scenes from nature. In order to increase the biodiversity on display, museums often prioritize the inclusion of different species over showing multiple specimens of the same kind (Schilthuizen et al., 2015). The Spectrum of Life Display in the Hall of Biodiversity at the American Museum of Natural History, for example, contains ~1,500 specimens, most of them representing unique species.

Yet, there are potential advantages to displaying multiple examples from the same category. When shown only a single category example, visitors may focus on irrelevant details. Displaying a pair of category members enables visitors to compare them, guiding attention toward relational structure. Indeed, children tend to sort objects in terms of taxonomic relations (e.g., vegetable) over perceptual features (e.g., round) after comparing two category members (Gentner and Namy, 1999). Displaying multiple category examples could also illuminate aspects of natural kinds that may otherwise be overlooked or misunderstood by visitors. For example, people often underestimate the variability that exists within biological categories (Shtulman and Schulz, 2008). Tigers may be thought to have about the same number of stripes, or ladybugs the same number of spots. Adults who underestimate within-category variation tend to have a poorer understanding of evolution (Shtulman and Schulz, 2008). Providing visitors with the opportunity to compare two category members that differ from one another—e.g., a tiger with many stripes vs. one with few—could help them appreciate the amount of variability that exists within biological categories.

**Figure 1:**
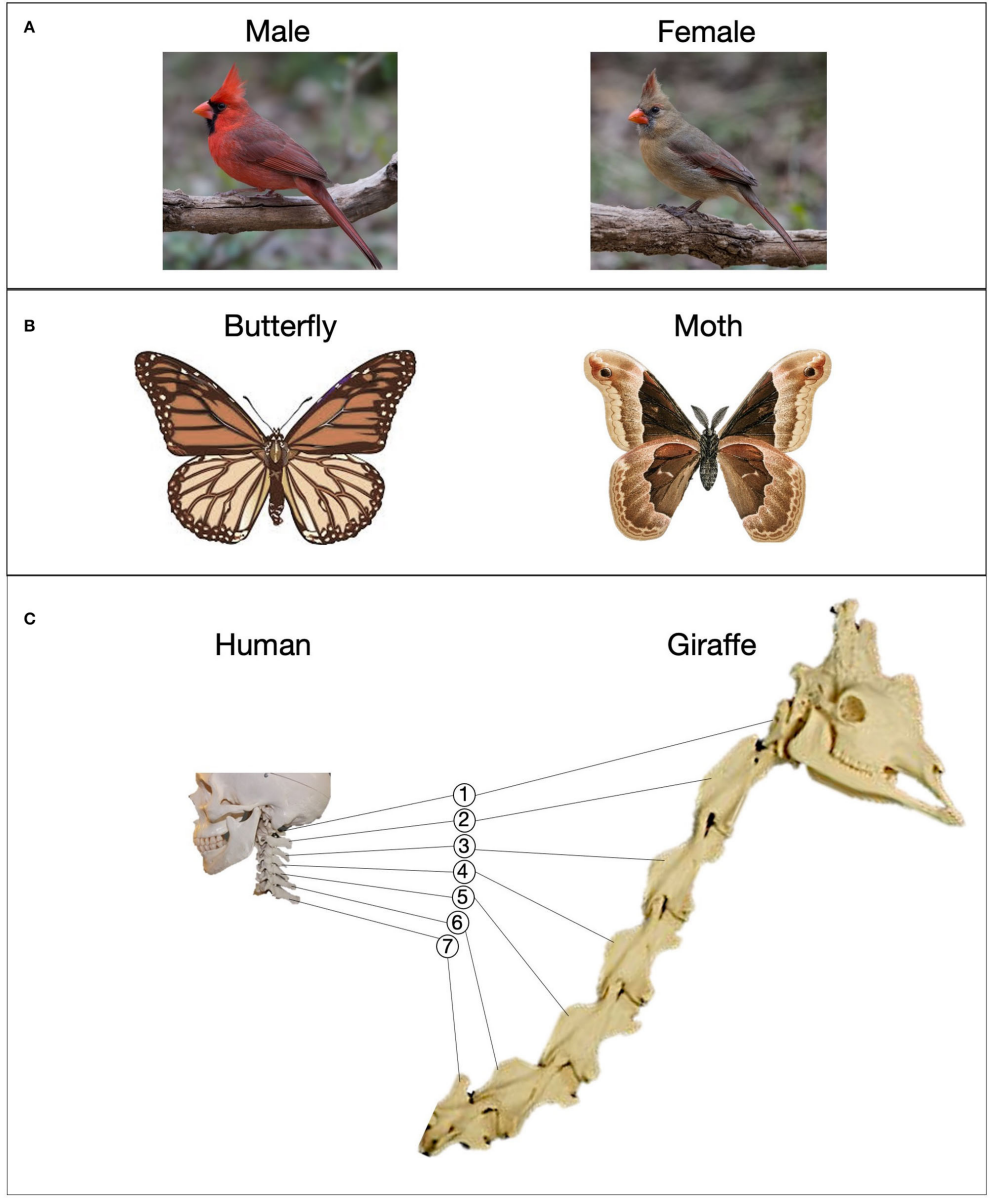
Examples of comparisons involving natural specimens. **(A)** A male and female northern cardinal. Photographs by Andy Morffew; **(B)** a butterfly and a moth; **(C)** the cervical vertebrae of a human and a giraffe.

Displaying multiple examples can also convey *systematic* variability within a category. For example, biological males and females of a species often have characteristically different traits, known as *sexual dimorphism*. Birds provide a number of striking examples. In northern cardinals, for instance, adult males have a bright red body and black coloring around the beak, whereas adult females are pale brown with reddish wings (Figure 1A). In other birds, like mandarin ducks and peacocks, the disparity in coloration is even greater. These within-category differences are driven by natural selection—in particular, females' preference for ornamental coloration in males (e.g., Hill, 2006). Yet, these interesting and informative patterns are effectively ignored when only a single specimen is on display. In fact, visitors may form a skewed impression, because male specimens are displayed approximately twice as often as female specimens (Mendenhall et al., 2020). The comparison of a male and female category member can draw attention to systematic variability, and, if multiple kinds of animal are compared, shed light on broader patterns in nature.

Paired examples can also help visitors distinguish between members of *different* categories. For example, students were better able to learn a geological structure, such as fault, when an example of the category was shown alongside a visually-similar image that did not contain this structure (Jee et al., 2013). Similarly, medical students learned to diagnose diseases from X-rays more accurately when a disease example was shown with a similar but healthy example as they learned (Kok et al., 2013). Encouraging visitors to compare specimens from different categories—*contrasting* cases—could help them to notice key taxonomic differences. This may be especially beneficial when two categories are readily confused. Figure 1B shows a display that includes a butterfly and a moth, two insects belonging to the order *Lepidoptera*. Although similar in appearance, butterflies and moths can be distinguished by several anatomical features, including their antennae (butterfly: thin with bulbed ends; moth: feathery/saw-edged), and body shape (butterfly: thin; moth: thick). These category-distinguishing features stand out when the two examples are directly compared. Contrasts can also promote relational concept learning. For example, Strouse and Ganea (2021) found that 3-year-old children were more likely to learn about camouflage from a storybook in which a camouflaged animal (light animal on light background) was compared with a similar, noncamouflaged animal (light animal on dark background).

Finally, pairs of specimens could be used to shed light on the deep, evolutionary connections between different species. For example, dolphins are very different from most other mammals in terms of their appearance and habitat. Displaying the bones of a dolphin flipper alongside those of a human hand permits a comparison that illuminates a remarkably similar skeletal structure. Along these lines, comparing the neck bones of a human and a giraffe (Figure 1C) reveals that each has the same number of cervical vertebrae (seven) despite dramatic differences neck length—a phenomenon known as *evolutionary stasis* (e.g., Williams, 1992). It can also be effective to have visitors compare a specimen against their own body. Callanan et al. (2016) found that children and adult museumgoers engaged in deeper conversations about a fossilized mammoth femur when the exhibit enabled a visitor to line up their own leg with the fossil. When the fossil was displayed in a case, visitors often merely labeled it a “bone.” When visitors could sit down next to the fossil to compare its massive size against their own leg, they used different terms, including the specific name of the bone (“femur”), and the extinct animal to which it belonged (“mammoth”).

Natural specimens can be paired in a number of ways in order to promote scientifically-informative comparisons. In addition to natural objects, museums often display scientific models that represent causal systems, such as plate tectonics, state changes of matter, and planetary motion. Comparisons between multiple models/visual representations could help visitors understand these models and make important connections to the real world.

## Exhibits that Promote Comparisons Involving Scientific Models

Scientific models—such as physical replicas and computer simulations—are representations of real-world systems (National Research Council, 2012). In such models, key elements of a system can be emphasized, and irrelevant details removed; objects that are imperceptibly small or large can be brought into view; events that unfold too quickly or too slowly to notice can be slowed down or sped up. These aspects of models can help to explain how the world works (Clement, 2008; Sibley, 2009; Jee et al., 2010; Kastens and Rivet, 2010; National Research Council, 2012; Stull and Hegarty, 2016). Models and modeling are therefore regarded as important crosscutting concepts in science education (National Research Council, 2012). Models are widespread in science museums—from reconstructions of extinct species to interactive simulations of natural systems and human-made machines. We focus on models of causal processes, especially those that depict real-world systems.

Like a good analogy, a good scientific model reflects the relational structure of the system it represents (Sibley, 2009). The classic science fair volcano—which erupts when vinegar and baking soda are poured into a crater at its top—is a poor scientific model, because it misrepresents the cause of a volcanic eruption. Of course, even well-designed models can be challenging to understand. Museum models that are highly abstract, with little resemblance to nature, can be difficult for visitors to grasp (Afonso and Gilbert, 2007). However, models that are highly realistic may obscure relevant properties or behaviors in a sea of trivial details (Uttal et al., 1997; Goldstone and Son, 2005; Kokkonen and Schalk, 2020). With a single model, it is hard to strike an ideal balance between emphasizing relational structure—which is central to deep learning—and retaining realistic surface details—which ground the model in the real world (Fyfe et al., 2014). Displays that enable visitors to compare a model with another model or visual representation could provide a way to overcome this challenge.

**Figure 2:**
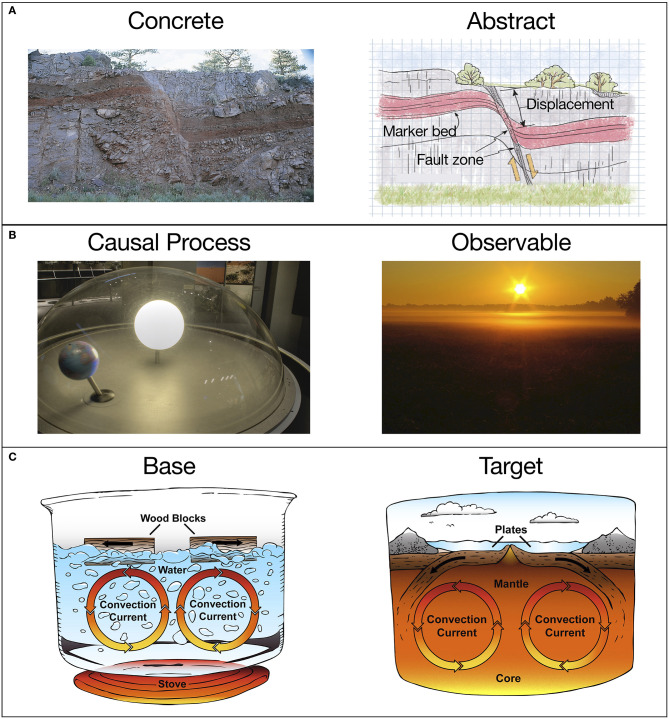
Examples of comparisons involving scientific models. **(A)** A concrete and abstract representation of a geological structure. Excerpted from Essentials of Geology, 3rd Edition. Copyright © 2009 by Stephen Marshak. Used with permission of the publisher, W. W. Norton & Company, Inc. All rights reserved. **(B)** a model solar system and an observation from Earth's surface; **(C)** a visual analogy between a boiling pot of water (base) and mantle convection (target).

Displaying multiple representations of the same object or system—one showing how the object or system appears in real life, and the other emphasizing relational structure—could be effective in many cases. For example, geological structures can be difficult for novices to distinguish in natural contexts (Jee et al., 2014). Showing a photo of a geological structure along with an abstract model of the structure, as in Figure 2A (from Marshak, 2009), can help students make the connection between realistic and abstract representations. Designing an exhibit such that a concrete/perceptually-rich model is shown *before* an abstract/idealized model may be especially helpful—a sequence known as *concreteness fading* (Goldstone and Son, 2005; Fyfe et al., 2014). Encountering a perceptually-rich representation first can help to ground and disambiguate the more abstract model that is seen later on. In terms of structural alignment, the concrete representation serves as the base, and the more abstract representation, the target (Kokkonen and Schalk, 2020). In a museum, concreteness fading could be accomplished in a number of ways—a digital display that transitions from concrete to abstract at the push of a button or slide of a bar, or using a projector to overlay a realistic image onto an abstract physical model, etc.

When scientific models depict imperceptible objects or events, such as molecular structures or planetary motion, it may be crucial to connect the model to objects and events that *can* be experienced in everyday life. Displaying the behavior of the model alongside a related observable event could allow visitors to better appreciate these connections. In a recent study, 3rd-grade students were instructed to compare a model of Earth rotation alongside a synced-up video of the Sun's apparent motion in the sky. This enabled the students to see how the sunrise we observe when a location on Earth becomes exposed to sunlight is due to our planet's eastward rotation. The students who could compare the modeled and observable events learned more about the cause of the day/night cycle than students who received lessons involving only the model (Jee and Anggoro, 2019). A similar approach could be implemented in a museum using a solar system model synced with a nearby display of the “sky” as in Figure 2B. As Earth rotates in the model solar system, the accompanying display shows the view from a location on Earth's surface. Other counterintuitive scientific ideas, such as the link between molecular activity and state changes of matter, could be facilitated through similar pairings of models and more familiar or intuitive visual representations (e.g., Samarapungavan et al., 2017; Stieff, 2019).

The use of multiple models/representations could also enhance cross-domain analogies—comparisons between examples from different subject areas. When a model represents an unfamiliar system (the target), an analogy to a familiar example (the base) can help visitors understand how the model works. For example, sound waves may be compared to ripples in a pond; the mitochondria of a cell may be compared to a power plant; the convection of Earth's mantle to a boiling pot of water, etc. Analogies like these are often used to communicate scientific ideas (e.g., Glynn, 1991; Harrison and Treagust, 2006; Jee et al., 2010; Holyoak and Lee, 2017; see also the crowd-sourced list of science analogies at https://tinyurl.com/wrcp725). In museum exhibits, analogies often appear in text form, such as a sign or caption for a display (Valle and Callanan, 2006). However, text-based analogies rely on a visitor's accurate recollection of the base domain, and also their ability to map the base and target. Both of these processes are resource-demanding and error-prone (Richland et al., 2007; Simms et al., 2018). Adding a visual representation of the base can help learners grasp an analogy, and reduce the cognitive burden of retrieval and mapping (Richland and McDonough, 2010). Indeed, 4th-grade students gained more knowledge from analogical instruction about scientific processes when both the base and target cases were visually represented as opposed to showing the target alone (Matlen et al., 2011). Figure 2C shows one of the visual analogies from Matlen and colleagues' study—a boiling pot of water (base) and mantle convection (target). When an exhibit includes cross-domain analogies such as this, adding a visual representation of the source example may help visitors perform the intended structural alignment.

## Exhibit-Based Supports for Comparison and Alignment

Exhibits that display multiple specimens, models, and other visual representations provide visitors with the opportunity to engage in scientifically-informative comparisons. Yet, visitors may require additional cognitive supports to fully benefit from these opportunities. In the informal learning context of a museum, it may be crucial to incorporate supports for comparison into the exhibit itself.

A number of exhibit-based supports could facilitate comparisons involving natural specimens and models. Surface similarity is one key factor. Superficially similar items are easier to align, and are helpful for initiating relational learning (Thompson and Opfer, 2010; Gentner et al., 2011; Jee et al., 2013). If the goal is to highlight differences, high-similarity contrasting items can draw a visitor's attention to the features that vary between the items (Gentner and Markman, 1994; Sagi et al., 2012; Strouse and Ganea, 2021). Though natural specimens are not entirely manipulable, it may be possible to select examples with high overall similarity to help visitors perform the intended comparison. The cardinals in Figure 1A, for example, are similar in many respects—size, orientation, background, etc.—which helps to draw attention to the difference in color. Likewise, the size, color, material, and other aspects of a model could be controlled to enhance its similarity to other representations in a display. For example, when a museum display included models of a stable and unstable building that were highly similar in height, color, construction materials, etc., 6–8-year-olds were more likely to learn the key feature of the stable building—a diagonal brace (Gentner et al., 2016). If increasing the perceptual similarity of the two models is not a viable option, comparison can be supported by adding a third example that is halfway between the two in terms of its appearance. The inclusion of an intermediate case establishes a bridging analogy that clarifies the connection between the more extreme pair (Clement, 1993).

Another consideration is how a pair of related items are arranged in space. To facilitate comparison, two objects should be placed in close proximity, and perhaps visually segregated from other items in a display, e.g., by placing a boundary around them. This spatial contiguity could help visitors to realize that a meaningful relationship exists, and makes it easier to examine the two cases simultaneously (Mayer and Moreno, 2003). Another spatial consideration is the relative placement of the items in the display (Richland et al., 2007; Richland and Simms, 2015; Matlen et al., 2020). For pairs of specimens or models with a vertical orientation (e.g., part A *above* part B, etc.), side-by-side placement is optimal for comparison (Matlen et al., 2020). For those with a horizontal orientation (part A *beside* part B, etc.), placing one above the other is optimal. In Figure 1C, for example, the human and giraffe pair are placed in optimal fashion for alignment—specimens with vertical orientation placed next to each other.

Spatial factors can also support relational thinking and learning from interactive exhibits. For exhibits in which visitors are invited to compare themselves against a museum specimen—a dinosaur's footprint, a condor's wingspan, a mammoth's leg bone, etc.—deeper learning is more likely to occur when the visitor can place their body in an optimal position for the alignment (Callanan et al., 2016). Exhibits that include levers, knobs, and buttons can be easier to understand and control when they conform to commonplace metaphors between space and quantity, such as “more is up” and “less is down” (Lakoff and Johnson, 1980; Allen, 2004). Spatial structure can also support reasoning about relational rules that govern a natural or artificial system. For example, 3-year-olds were more likely to infer a relational rule—e.g., that two of the same objects were needed to activate a machine—when the two objects were inserted into openings at either side of the machine (highlighting their relation) as opposed to being placed on top of it (Walker et al., 2020).

Language provides another useful support for comparison. Labels, captions, and other verbal information can clarify connections between examples. Even young children benefit from verbal prompts to compare, and learn abstract relational categories more efficiently when category members are labeled with the same term (Waxman and Markow, 1995; Gentner and Namy, 1999; Gentner et al., 2011). When causal processes are displayed in multiple visual representations, children learn more when prompted to think about the relationships between the representations (Hansen and Richland, 2020). Labels, captions, instructions, etc. can also benefit pre-literate children by influencing how their older caregivers behave at an exhibit. Simple signs in a display can help parents appreciate the educational value of museum exhibits (Song et al., 2017). This awareness could lead caregivers to capitalize on educational opportunities that they and their child might otherwise miss.

When children engage in an exhibit together with an adult caregiver, they tend to demonstrate more critical scientific thinking, such as comparing different sources of evidence (Crowley et al., 2001). Parents may produce spontaneous analogies to help their children grasp the scientific ideas they encounter (Valle and Callanan, 2006). Children also learn more when their parent uses language that highlights key features or relations—e.g., referring to diagonal bracing (“angle,” “brace,” “cross-beam,” “diagonal”) while building a model tower (Gentner et al., 2016). Parents' nonverbal cues, such as gestures, could also help to draw attention to relevant relationships and support the alignment process (Alibali et al., 2013; Richland, 2015).

Labels and other text may be most effective when placed within a display, in close proximity to related specimens and models, as opposed to outside the display on a placard—another application of the spatial contiguity principle (Mayer and Moreno, 2003). This proximity can help to ensure that visitors notice the verbal information *before* engaging with the exhibit materials. Indeed, parents' use of causal language predicted their children's productive use of exhibit materials (e.g., building machines with gears) only if it occurred before children used the materials (Callanan et al., 2019).

## Further Thoughts on Comparison and Science Learning

Our discussion of comparison emphasized the educational potential of promoting an underutilized cognitive tool through the design of museum displays. As we move to test our comparison-based approach in a museum setting, we recognize that designing a successful exhibit involves numerous considerations besides meeting educational goals. Children's museum exhibits should be interesting and entertaining, they should engage visitors at different age levels who may interact with an exhibit alone or together (Rigney and Callanan, 2011), and they should be accessible to children and adults with diverse backgrounds and abilities (Shaby et al., 2016). Ideally, methods that promote informative comparisons in a museum will enhance children's thinking and learning without sacrificing their enjoyment, exploration, and engagement. Indeed, without willful engagement, visitors have little chance of benefiting from even the most effective visual displays. Thus, to better evaluate the comparison-based approach, we must use metrics relevant to museum exhibit practitioners, such as tracking visitor groups' time spent at the exhibit, their verbal and nonverbal references to the materials in an exhibit, and the roles that caregivers take in supporting children's learning (Crowley et al., 2001; Haden et al., 2016; Callanan et al., 2017; Horn, 2018).

Though our focus is on museum-based learning, methods that promote relational thinking could be applied broadly in education. Research on math and science learning has revealed that relatively small changes to existing materials—such as the number and type of practice problems that students receive, or the spatial layout of a science diagram—can make a difference in students' mastery of the material (Higgins, 2020). Encouragingly, when an instructional sequence sets the stage for structural alignment, children can recognize and transfer relational structure without explicit guidance (Sidney, 2020). By further incorporating cognitive supports into educational materials and lessons, remote and independent student learning—which have soared during the COVID-19 pandemic—could be made more effective and manageable for teachers, students, and caregivers. More broadly, this approach would make science more accessible for young children who may otherwise receive little instructional support at home or at school.

## Data Availability Statement

The original contributions presented in the study are included in the article/supplementary material, further inquiries can be directed to the corresponding author/s.

## Author Contributions

BJ and FA share first authorship in light of their comparable contributions to this project. Both authors approved the final version of the manuscript for submission.

## Conflict of Interest

The authors declare that the research was conducted in the absence of any commercial or financial relationships that could be construed as a potential conflict of interest.
